# Influence of a Very High-Molecular Weight Fucoidan from *Laminaria hyperborea* on Age-Related Macular Degeneration-Relevant Pathomechanisms in Ocular Cell Models

**DOI:** 10.3390/md23030101

**Published:** 2025-02-25

**Authors:** Philipp Dörschmann, Georg Kopplin, Tabea Thalenhorst, Charlotte Seeba, Sadia Fida Ullah, Vaibhav Srivastava, Johann Roider, Alexa Klettner

**Affiliations:** 1 Department of Ophthalmology, University Medical Center, University of Kiel, Arnold-Heller-Str. 3, Haus 25, 24105 Kiel, Germanyjohann.roider@uksh.de (J.R.); alexa.klettner@uksh.de (A.K.); 2 Alginor ASA, Haraldsgata 162, 5525 Haugesund, Norway; georg@alginor.no; 3 Division of Glycoscience, Department of Chemistry, School of Engineering Sciences in Chemistry, Biotechnology and Health, Royal Institute of Technology (KTH), AlbaNova University Centre, SE106 91 Stockholm, Sweden; ullahsf@kth.se (S.F.U.); vasri@kth.se (V.S.)

**Keywords:** sulfated fucan, fucoidan, retinal pigment epithelium-specific 65 kDa protein (RPE65), vascular endothelial growth factor (VEGF), phagocytosis, gene expression, protectin (CD59), trans-epithelial electrical resistance (TEER), interleukin, toll-like receptor, polyinosinic/polycytidylic acid (PIC)

## Abstract

Fucoidans from *Laminaria hyperborea* (LH) can be antioxidative, antiangiogenic, and anti-inflammatory. In this study, a very high-molecular weight (3700 kDa) fucoidan from LH, FucBB04, was tested regarding its bioactivity in age-related macular degeneration (AMD) models *in vitro*. Primary retinal pigment epithelium (RPE) from pig eyes, human uveal melanoma cell line OMM-1, and RPE cell line ARPE-19 were used. Substituents of the extract were determined with chemical analysis. Cell viability was tested with tetrazolium assay (MTT), oxidative stress was induced by H_2_O_2_ or erastin, respectively. Secreted vascular endothelial growth factor A (VEGF-A) was assessed with ELISA. Retinal pigment epithelium 65 kDa protein (RPE65) and protectin (CD59) protein expression were tested in Western blot. Cell barrier was assessed by measuring trans-epithelial electrical resistance (TEER), phagocytic ability by a fluorescence assay. Gene expression and secretion of interleukin 6 (IL-6) and interleukin 8 (IL-8) were tested in real-time PCR and ELISA. FucBB04 displayed no oxidative stress protective effects. Its effect on VEGF was inconsistent, with VEGF secretion reduced in primary RPE, but not in ARPE-19. On the other hand, Lipopolysaccharide (LPS) and polyinosinic/polycytidylic acid (PIC)-induced IL-6 or IL-8 secretion was reduced by FucBB04, while complement inhibiting protein CD59 was not affected. In addition, FucBB04 did not influence the gene expression of IL-6 or IL-8. Visual cycle protein RPE65 expression, phagocytic ability, and barrier function were reduced by FucBB04. Very high-molecular weight fucoidan from LH shows bioactivities against AMD-related pathological pathways, but adverse effects on RPE function may limit its suitability as a therapeutic compound. Smaller high-molecular weight fucoidans are recommended for further research.

## 1. Introduction

Age-related macular degeneration (AMD) is the major cause for blindness and severe visual impairment in the elderly in the industrialized world [1]. The disease can be divided in an asymptomatic early and two vision threatening late forms. The late forms consist of the atrophic or “dry” form, in which degenerate changes in the retinal pigment epithelium (RPE) and the photoreceptors result in a slow progressive vision deterioration, and the neovascular exudative or “wet” form, in which vessels grow under or into the retina and visual acuity decreases rapidly. Currently, only the late wet form can be targeted by therapeutics (mainly intravitreal VEGF inhibitors), which may hold or reduce disease progression. New therapeutics targeting the early forms and slowing the progression are clearly warranted [2].

On a cellular level, the pathology of AMD occurs in the photoreceptor/RPE/choroid complex, with the RPE being the major player in AMD development [3]. The RPE exerts many functions in the retina, supporting the photoreceptors and upholding visual function. RPE cells protect the photoreceptors by scavenging stray light und protecting against oxidative stress [4]. They phagocytose shed photoreceptor outer segments and participate in recycling of the visual pigment [5]. Furthermore, they form the outer blood–retina barrier, thereby regulating transport from and to the photoreceptors and contributing to the retinal immune privilege [6]. On the other hand, they can contribute to inflammation, as they act as sentinels to danger signals, expressing toll-like receptors (TLR) 3 and 4, and secreting various pro-inflammatory stimuli upon activation [7,8].

On the cellular and tissue level, the pathomechanisms in AMD development include oxidative stress, lipid dysregulation, inflammation, complement activation, and, in the wet form, angiogenesis caused by increased VEGF secretion [8,9,10,11]. A compound that could target several of these mechanisms would be of high interest for treating early AMD development and slowing down or halting its progression.

Such a potential compound is fucoidan, a polysaccharide obtained from brown seaweed [12]. Our group has previously shown that fucoidans could exert antiangiogenic, antioxidative, and anti-inflammatory effects in retinal cells [13,14,15]. However, fucoidans are a heterogenic group and their bioactivity depends strongly on its species of origin and the extraction methods, as well as other factors [16]. In particular, the pro- and antiangiogenic properties of fucoidan have been linked to their molecular weight, with low-molecular weight fucoidans described as pro- and high-molecular weight fucoidans as antiangiogenic [17]. In addition, anti-inflammatory and complement-inhibiting properties of fucoidans have been related to their degree of sulfation and their fucose content [18,19]. Our own studies support these findings, as beneficial bioactivities of fucoidans regarding AMD-related pathways are connected to a higher molecular weight, a higher degree of sulfation, and a high content of fucose [13].

Previously, we could show that fucoidan from *Laminaria hyperborea* (LH) with a high-molecular weight (1549 kDa) showed promising characteristics compared to fucoidans of lower molecular weight from the same species [20]. Therefore, we wanted to investigate the effect of very high-molecular weight fucoidan (3700 kDa; FucBB04) from this species.

We investigated the bioactivity in regards to the important pathomechanisms of oxidative stress, inflammation, and angiogenesis (VEGF secretion) in ocular *in vitro* models, using the human RPE cell line ARPE-19 [21], primary porcine RPE cells [22], and the uveal melanoma cell line OMM-1 [23]. Primary porcine RPE cells are an excellent model for human RPE cells, displaying a highly differentiated phenotype (when used without further passaging) and a high barrier (as assessed in transepithelial electric resistance), exhibiting natural functions such as phagocytosis, sentinel functions, and cytokine secretion [24]. ARPE-19 cells are an RPE cell line with some similarities to RPE cells, such as a constitutive (albeit lesser) secretion of VEGF. Furthermore, concerning angiogenesis or cell death, they exhibit similar gene expression while being more susceptible to oxidative stress than fully differentiated RPE cells, rendering them a good model for testing oxidative stress protection [24,25]. OMM-1, an uveal melanoma cell line, was added as they are more susceptible to oxidative stress compared to primary RPE or ARPE-19 cells [26].

In addition to testing AMD-relevant bioactivity, we investigated the effect of this fucoidan on important functions of the RPE, assessing survival, phagocytosis, barrier properties, and the expression of a protein important for the visual cycle (retinal pigment epithelium 65 kDa protein, RPE65) [27] and complement inhibition (protectin, CD59) [28]. Furthermore, we present the highest molecular weight fucoidan reported so far.

## 2. Results

### 2.1. Chemical Characterization of Fucoidan

The applied extraction and purification methods omitted depolymerization and further removed lower molecular weight fractions (300 kDa molecular weight cut-off, MWCO) from the sample, aiming for high-molecular weight fucoidan from LH.

Monosaccharide analysis of FucBB04 revealed a fucose content of 91.59 ± 1.40%, classifying it as a fucan according to Deniaud-Bouët et al. (2017) [29]. Galactose (0.50 ± 0.42%), xylose (0.71 ± 0.05%), rhamnose (1.32 ± 0.26), mannose (0.44 ± 0.15), and three uronic acids, namely galacturonic acid (1.60 ± 0.12), mannuronic acid (1.95 ± 0.23), and guluronic acid (1.86 ± 0.08), were found (Table 1). Even though monosaccharide residues of xylose, rhamnose, and uronic are common in fucoidans from brown seaweed [30,31], this is, to our knowledge, the first time those are reported for fucoidan from LH [32,33].

Mass spectrometry of FucBB04 revealed a sulfur content (S) of 12.4%, corresponding to a sulfate content (NaSO_3_) of 39.5% and a sulfation degree (DS) of approximately 0.95 [33] (Table 2). Raman spectra showed strong bands at 1063 cm^−1^ and 839 cm^−1^, related to vibrations of the sulfate group, confirming sulfation in the C-4 position [34].

**Figure 1:**
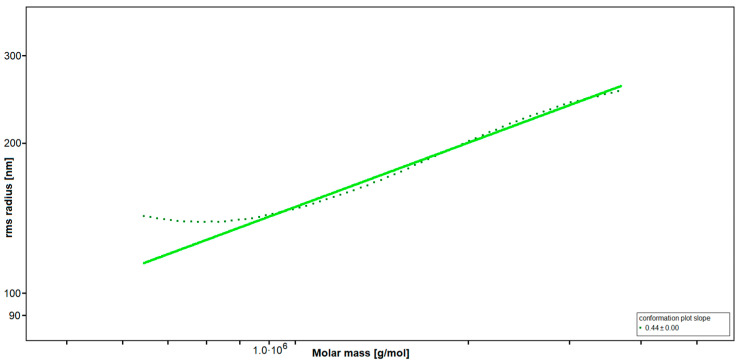
Rms conformation plot of the high-molecular weight fucoidan (FucBB04) giving the rms (root mean square) radius [nm] versus M [g/mol]. The slope (b) = 0.44 indicates a random coil with substantial branching.

SEC-MALS (Size-Exclusion Chromatography with Multi-Angle Light Scattering) revealed a very high-molecular weight average of M_w_ = 3700 kDa, resulting in an average degree of polymerization (DP_n_) of 10,300, with an approximated average monosaccharide unit weight of 243 g/mol. To our knowledge, this is the highest molecular weight average for fucoidan yet reported [35,36]. The radius of gyration as the Z-average (R_z_) was found to be 249.0 nm. The overall shape of the molecule was determined through an rms conformation plot (root mean square radius [nm] versus M [g/mol], Figure 1), displaying a slope (b) of 0.44 and thus placing the overall shape of the molecule between random coil and sphere conformation (sphere b = 0.33; random coil b = 0.5; rigid rod b = 1) [37,38]. A high degree of branching for fucoidan from LH was previously reported [33]. However, light scattering experiments suggested an oligomeric branching ‘short-chain branches’, while a conformation towards a spherical shape is indicative of polymeric branching ‘long-chain branches’ or hyper-branching, respectively [39,40].

Total phenolic content (TPC) analysis using the Folin–Ciocalteu TPC assay as well as Raman spectroscopy showed absence of any phenolic residues within the sample.

### 2.2. Oxidative Stress Assays

In the development of AMD, oxidative stress is a major factor [41]. The retina is burdened with a high life–long exposure to oxidative stress because of high oxygen pressure, constant light exposure, high mitochondrial metabolism, lipid peroxidation, and intracellular deposits such as lipofuscin [41].

**Figure 2:**
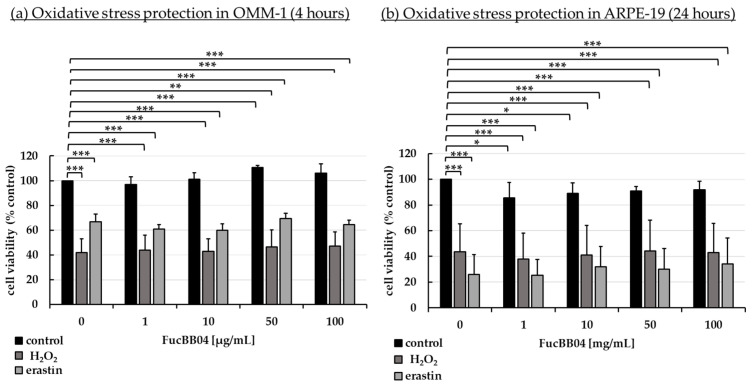
Oxidative stress assay. Human uveal melanoma cell line OMM-1 (**a**) and human retinal pigment epithelium cell line ARPE-19 (**b**) were treated with 1, 10, 50, or 100 µg/mL FucBB04 fucoidan. For oxidative stress insult, 500 µM H_2_O_2_ or 25 µM erastin for OMM-1 and 250 µM H_2_O_2_ or 20 µM erastin for ARPE-19 were applied for 4 h or 24 h, respectively. Cell survival was tested with tetrazolium bromide assay (MTT). Absorption was set into relation to the untreated control (100%). Data were normally distributed (Shapiro–Wilk test). One-sample *t*-test was conducted. * *p* ≤ 0.05, ** *p* ≤ 0.01, *** *p* ≤ 0.001 (compared to control, 0 µg/mL FucBB04 = 100%), *n* ≥ 5.

Established oxidative stress models were utilized as previously described [26,42,43]. Human uveal melanoma cell line OMM-1 (Figure 2a) and human RPE cell line ARPE-19 (Figure 2b) were stimulated with 1, 10, 50, or 100 µg/mL LH fucoidan FucBB04, respectively, for 30 min. In addition, oxidative stress insult was applied by 500 µM H_2_O_2_ or 25 µM erastin for OMM-1 and 250 µM H_2_O_2_ or 20 µM erastin for ARPE-19 for 4 or 24 h, respectively. Tetrazolium bromide assay [3-(4,5-dimethylthiazol-2-yl)-2,5-diphenyltetrazolium bromide, MTT] was used to determine cell viability. Absorption was set into relation to the untreated control, set as 100%. Regarding short-term effects of fucoidan on cell viability, FucBB04 increased the viability signal to 111 ± 2% (*p* = 0.0001) in OMM-1 and decreased the viability slightly in all concentrations in ARPE-19. These small effects should be of little biological relevance. Concerning oxidative stress assays, in OMM-1, viability was reduced to 42 ± 11% with 500 µM H_2_O_2_ (*p* = 0.0005) and to 67 ± 6% with 25 µM erastin (*p* = 0.0001), after 4 h of stimulation. Cell viability was not relevantly influenced with co-treatment of the extract. In ARPE-19, cell viability was decreased by 250 µM H_2_O_2_ to 44 ± 22% (*p* = 0.0002) and by 20 µM erastin to 26 ± 16% (*p* = 0.0001) after 24 h of stimulation. Also, the extract did not show any relevant effects concerning oxidative stress protection. Taken together, these results indicate that FucBB04 lacked short-term antioxidative effects in the models tested.

### 2.3. Vascular Endothelial Growth Factor Secretion

**Figure 3:**
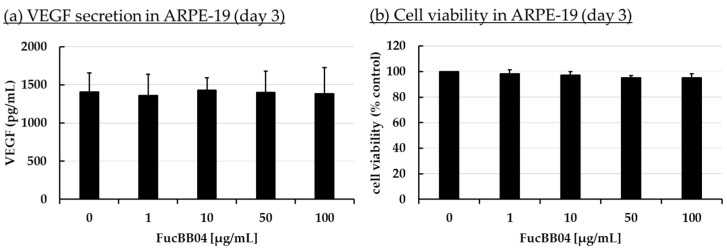
VEGF secretion and cell viability of ARPE-19. ARPE-19 cells were stimulated with 1, 10, 50, and 100 µg/mL FucBB04, respectively, for three days. Supernatant was collected for 24 h by renewing media with extracts on day 2. VEGF-A was determined in ELISA (**a**). MTT assay was performed at day 3 to measure cell viability (**b**). VEGF content in pg/mL of supernatants is shown. Data showed Gaussian distribution (Shapiro–Wilk test) and ANOVA by post hoc Dunnett’s test was performed to calculate significances. No significant changes compared to 0 µg/mL FucBB04 were detected. *n* = 4.

**Figure 4:**
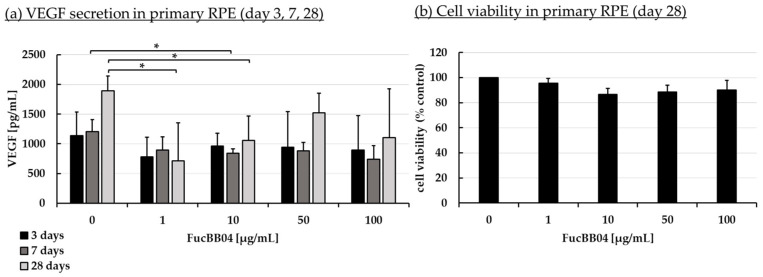
VEGF secretion and cell viability of primary RPE. Primary porcine RPE cells were stimulated with 1, 10, 50, and 100 µg/mL FucBB04 for 3, 7, and 28 days, respectively. Supernatant was collected on the individual days after changing media with extracts four hours before supernatant collection. VEGF-A was detected in ELISA (**a**). MTT assay was performed at day 28 to measure cell viability (**b**). VEGF content in pg/mL of supernatants is shown. Data showed Gaussian distribution (Shapiro–Wilk test) and ANOVA by post hoc Dunnett’s test was performed to calculate significances. * *p* ≤ 0.05 (compared to 0 µg/mL FucBB04 of the same day of treatment). *n* = 3.

Increased VEGF secretion plays an important role in the development of wet AMD [44]. Fucoidans have been shown to decrease VEGF in the supernatant of ocular cells [13]. ARPE-19 (Figure 3) and primary porcine RPE (Figure 4) cells were stimulated with FucBB04 (1, 10, 50, and 100 µg/mL), with the former one for 3 days and the latter for 3, 7, and 28 days. As primary RPE resemble the in vivo situation more closely, more time points and long-term effects were examined. Also, they produce nearly four times more VEGF per hour than ARPE-19 (ARPE-19: 66 pg/h and RPE: 243 pg/h, [20]). Supernatants were harvested after 24 h collection time for ARPE-19 and 4 h collection time for RPE. Supernatants were applied in human VEGF-A DuoSet ELISA. MTT assay was conducted at the end of the experiments to control for potential toxic effects influencing VEGF secretion.

In ARPE-19, no antiproliferative effects were found after three days (Figure 3b). VEGF secretion was also not significantly influenced (Figure 3a). In primary RPE cells, neither cell viability (Figure 4b) nor VEGF secretion (Figure 4a) was significantly altered by FucBB04 treatment after 28 days. Of note, a reduction is seen in VEGF content which does not reach statistical significance because of high standard deviation. After three days of treatment, all tested concentrations reduced VEGF slightly but not significantly. After seven days of stimulation, 10 µg/mL FucBB04 reduced VEGF content significantly from 1206 ± 202 pg/mL to 841 ± 73 (*p* = 0.0422). Here, 1, 50, and 100 µg/mL fucoidan reduced VEGF as well but reached no statistical significance. After 28 days of stimulation, secreted VEGF was significantly lowered by 1 and 10 µg/mL fucoidan from 1892 ± 248 pg/mL to 711 ± 640 pg/mL (*p* = 0.0407) and 1057 ± 411 pg/mL (*p* = 0.0394), respectively, while 50 and 100 µg/mL fucoidan also reduced it numerically without reaching significance.

### 2.4. Interleukin Secretion

**Figure 5:**
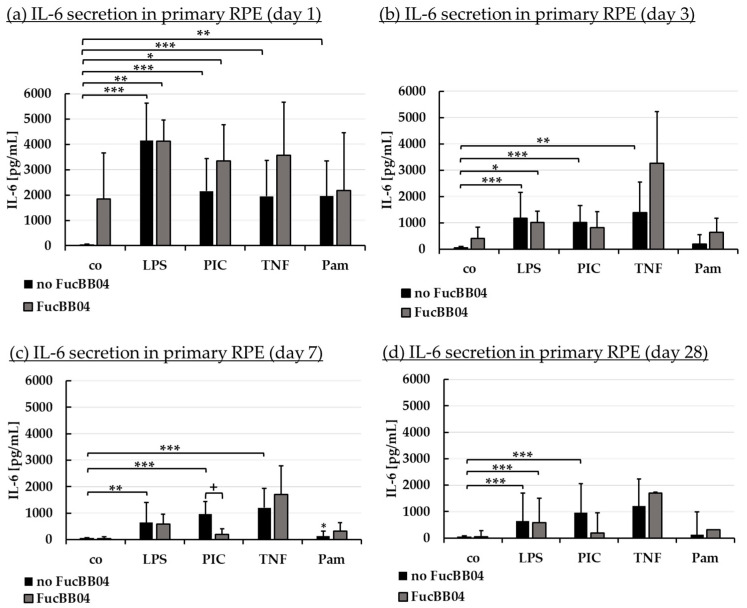
Interleukin 6 secretion. Primary porcine RPE cells were stimulated with 50 µg/mL FucBB04, and/or 1 µg/mL lipopolysaccharide (LPS), 10 µg/mL polyinosinic/polycytidylic acid (PIC), 50 ng/mL tumor necrosis factor alpha (TNF), or 10 ng/mL Pam2CSK4 (Pam) for 1 (**a**), 3 (**b**), 7 (**c**), and 28 days (**d**). Supernatants were collected and IL-6 was determined in ELISA. Data showed Gaussian distribution (Shapiro–Wilk test) and ANOVA by post hoc Dunnett’s test was performed to calculate significances. * *p* ≤ 0.05, ** *p* ≤ 0.01, *** *p* ≤ 0.001 (compared to co, no FucBB04); + *p* ≤ 0.05 (compared to stress control LPS, PIC, TNF or Pam, no FucBB04); co = control, *n* ≥ 3.

**Figure 6:**
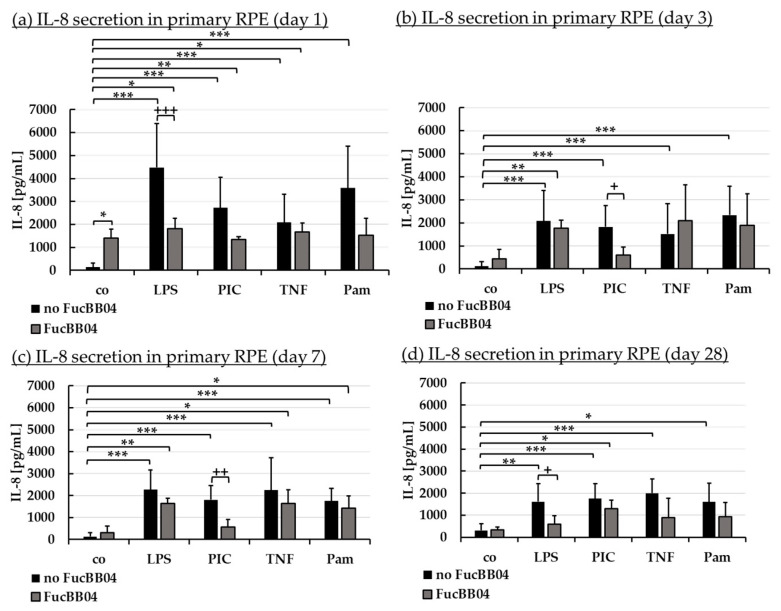
Interleukin 8 secretion. Porcine RPE cells were stimulated with 50 µg/mL FucBB04, and/or 1 µg/mL lipopolysaccharide (LPS), 10 µg/mL polyinosinic/polycytidylic acid (PIC), 50 ng/mL tumor necrosis factor alpha (TNF), or 10 ng/mL Pam2CSK4 (Pam) for 1 (**a**), 3 (**b**), 7 (**c**), and 28 days (**d**). Supernatants were collected and IL-8 was determined in ELISA. Data showed Gaussian distribution (Shapiro–Wilk test) and ANOVA by post hoc Dunnett’s test was performed to calculate significances. * *p* ≤ 0.05, ** *p* ≤ 0.01, *** *p* ≤ 0.001 (compared to co, no FucBB04); + *p* ≤ 0.05, ++ *p* ≤ 0.01, +++ *p* ≤ 0.001 (compared to stress control LPS, PIC, TNF or Pam, no FucBB04); co = control, *n* ≥ 3.

Inflammation plays an important role in the development of AMD and we have previously shown fucoidans to exert anti-inflammatory effects on RPE cells [9,14]. Primary RPE were treated with FucBB04 for 30 min, followed by pro-inflammatory stimulation by applying 1 µg/mL lipopolysaccharide (LPS), 10 µg/mL polyinosinic/polycytidylic acid (PIC), 50 ng/mL tumor necrosis factor alpha (TNF), or 10 ng/mL Pam2CSK4 (Pam), respectively. These agents and concentrations are based on previous findings [7]. Appropriate controls with fucoidan or inflammatory stimuli were included. Cells were stimulated for 1, 3, 7, and 28 days and supernatant collected for 24 h. Supernatants were analyzed in ELISA for porcine IL-6 (Figure 5) and IL-8 (Figure 6). Also, MTT assays were performed to determine cell viability after 1, 3, 7, and 28 days of treatment. No significant or relevant antiproliferative effects regarding stressors or extracts were found (refer to Appendix A.1).

Regarding IL-6 secretion, significant secretion was induced by all agents, with LPS and PIC exhibiting significant secretion at any time point tested. Pam only exhibited a short time response, losing this effect from day 3 (Figure 5b), and TNF no longer displayed a significant increase at day 28 (Figure 5d). FucBB04 alone did not induce a significant IL-6 release, but it was nominally increased at day 1 (Figure 5a) to 1846 pg/mL (*p* = 0.178). In general, FucBB04 displayed little effect on the level of secreted IL-6, with the exception of IL-6 secretion at day 7 (Figure 5c) after stimulation with PIC. Here, FucBB04 significantly reduced the secretion from 958 ± 485 pg/mL to 194 ± 210 pg/mL IL-6 (*p* = 0.001). Of note, several pro-inflammatory activations by different stimuli lost their significance after treatment with FucBB04, and for PIC treatment it was also numerically decreased on day 3 and day 28.

**Figure 7:**
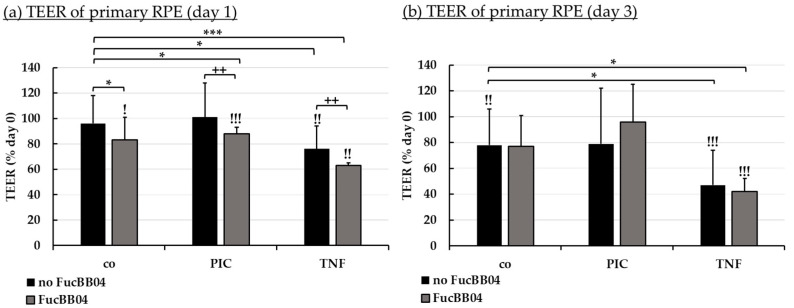
Barrier function of RPE. Porcine RPE cells cultured on transwell plates were treated with 50 µg/mL FucBB04 and/or 10 µg/mL polyinosinic/polycytidylic acid (PIC) or 50 ng/mL tumor necrosis factor α (TNF), respectively. Barrier function was determined by measuring the transepithelial electrical resistance (TEER) in Ω·cm². TEER was measured before stimulation (day 0), and after 1 (**a**) and 3 (**b**) days. TEER was calculated relatively to values of day 0 in percent. Data were normally distributed and one-sample *t*-test was conducted. * *p* ≤ 0.05, *** *p* ≤ 0.001 (compared to co, no FucBB04); ! *p* ≤ 0.05, !! *p* ≤ 0.01, !!! *p* ≤ 0.001 (compared to initial measurement of the same well); ++ *p* ≤ 0.01 (compared to stress control PIC or TNF, no FucBB04); co = control, *n* = 4.

Regarding IL-8 secretion, FucBB04 increased IL-8 release significantly on day 1 (Figure 6a) with 1396 ± 396 pg/mL (*p* = 0.032) compared to control with 135 ± 177 pg/mL. All pro-inflammatory stimuli significantly increased IL-8 secretion on all tested time points. FucBB04 reduced IL-8 in several stimulations: On day 1 and 28 (Figure 6d), LPS-induced IL-8 secretion was reduced by FucBB04 from 4475 ± 1924 pg/mL to 1809 ± 460 pg/mL (*p* = 0.0009), and from 1614 ± 813 pg/mL to 595 ± 381 pg/mL (*p* = 0.023), respectively. On day 3 (Figure 6b) and 7 (Figure 6c), PIC-induced IL-8 secretion was reduced from 1827 ± 920 pg/mL to 610 ± 335 pg/mL (*p* = 0.016), and from 1804 ± 656 pg/mL to 566 ± 345 pg/mL (*p* = 0.009), respectively. Taken together, FucBB04 seems to interact with LPS-induced TLR-4 activation at short-term and long-term conditions and on PIC-induced TLR-3 activation at mid-term conditions, indicating an anti-inflammatory potential. There are clear differences in the activity of fucoidan between IL-6 and IL-8 assays as well as for the inflammatory agents which excludes a mere scavenging effect.

### 2.5. Interleukin Gene Expression

In addition to secreted proteins, we also investigated IL-6 and IL-8 gene expression. We focused on PIC-stimulated RPE cells (as they showed the most promising activities in Section 2.4) for three and seven days with 50 µg/mL FucBB04 and/or 10 µg/mL PIC. RNA was isolated, transcribed to cDNA, and real-time polymerase chain reaction was conducted to measure relative gene expression of IL-6 (*IL6*) and IL-8 (*CXCL8* or *IL8*). Glyceraldehyde-3-phosphate dehydrogenase (*GAPDH*) was used as endogenous control.

PIC stimulation did not significantly increase *IL6* or *IL8* gene expression after three days (Table 3), in contrast to the protein assays. *IL8* expression of control was more than two times lower the PIC (Rq = 0.434), but reached no significance. FucBB04 alone displayed no effect on *IL6* or *IL8* gene expression. *IL6* gene expression induced by PIC in the presence of fucoidan was numerically lower than PIC alone (Rq = 0.603), which, however, did not reach significance. Regarding seven days of gene expression, PIC stimulation led to an increased *IL6* and *IL8* gene expression (Table 4), which did not reach statistical significance. Additional treatment with FucBB04 did not display any significant influence on PIC-induced *IL6* or *IL8* gene expression.

### 2.6. Barrier Measurement

The manifestation of a cell barrier is an important task of RPE, establishing the outer blood–retina barrier [45]. The influence of FucBB04 and pro-inflammatory agents on RPE barrier function was tested. Primary porcine RPE were cultivated on transwell plates. Immediately before treatment (day 0), transepithelial electrical resistance (TEER) was measured, and cells were treated with 50 µg/mL FucBB04 and/or 10 µg/mL PIC or 50 ng/mL tumor necrosis factor alpha (TNF). PIC was chosen regarding possible protective effects of fucoidans (Section 2.4 [43]) and TNF as a known barrier-reducing agent in the RPE [7,46]. After 1 and 3 days of stimulation, TEER was measured again and set in relation to day 0 values in percent (Figure 7). Significances were calculated compared to control with no FucBB04, to day 0 values of the same well, and to stress controls PIC or TNF with no FucBB04.

After one day (Figure 7a), barrier function was reduced compared to control and to the day before treatment with FucBB04, FucBB04 plus PIC, TNF and FucBB04 plus TNF, but not PIC alone. Also, barrier function was significantly stronger decreased by FubBB04 treatment compared to stress control. Fucoidan reduced TEER after PIC treatment from 101% ± 21% to 88% ± 5% (*p* = 0.0035) when FucBB04 was co-applied, and after TNF treatment from 76% ± 18% to 63 ± 2% (*p* = 0.0027) when FucBB04 was co-applied. After three days (Figure 7b), the negative effects of fucoidan on the barrier are lost, while control and TNF-related treatments are reduced compared to TEER before treatment.

### 2.7. Protein Expression

**Figure 8:**
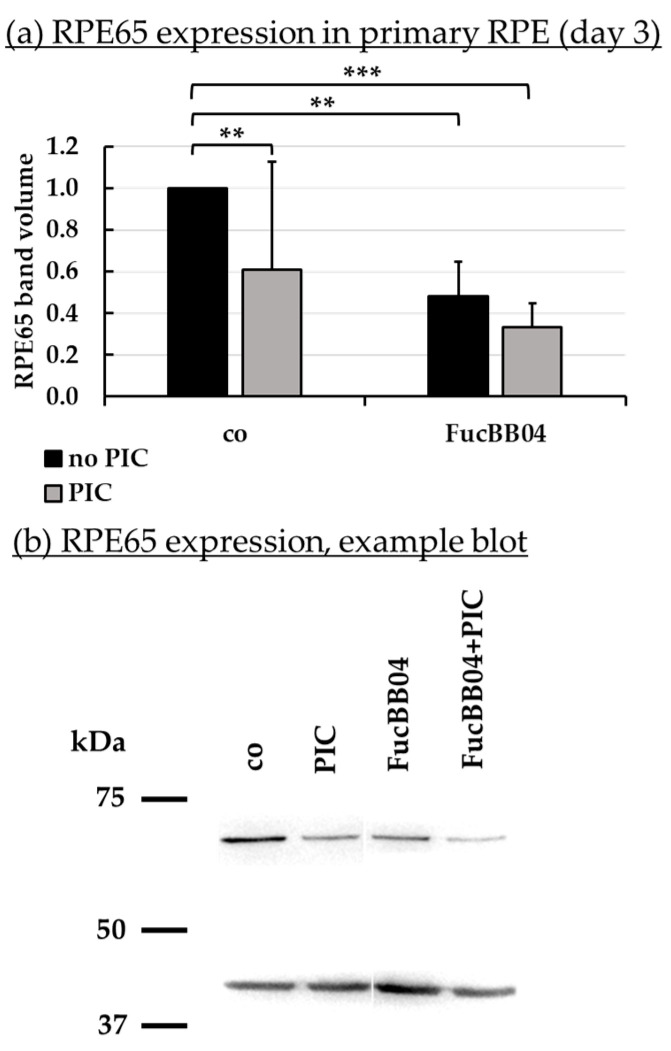
RPE65 protein expression. Primary porcine RPE cells were treated with 50 µg/mL FucBB04 and/or 10 µg/mL polyinosinic/polycytidylic acid (PIC) for three days. RPE65 expression was determined in Western blot (exemplary blot in (**b**)). β-actin (48 kDa) expression was used for normalization. Band volumes were calculated in TotalLab (**a**) in relation to untreated control. Data were normally distributed and one-sample *t*-test was conducted. ** *p* ≤ 0.01, *** *p* ≤ 0.001 (compared to co, no PIC); co = control, *n* = 5.

**Figure 9:**
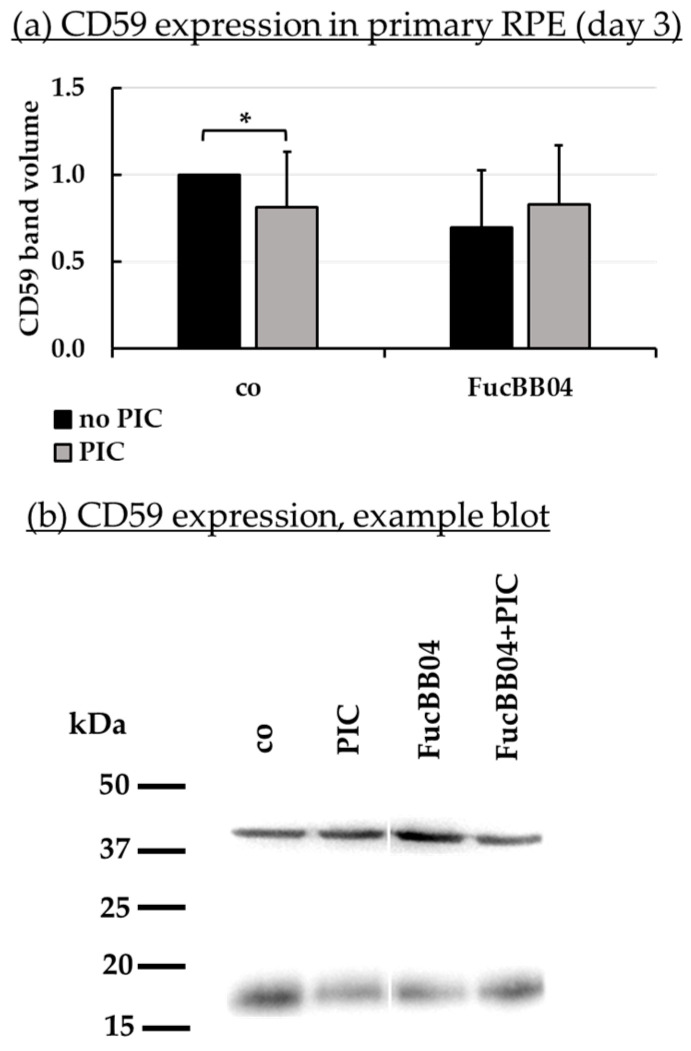
CD59 protein expression. Primary porcine RPE cells were treated with 50 µg/mL FucBB04 and/or 10 µg/mL polyinosinic/polycytidylic acid (PIC) for three days. Expression of CD59 was determined in Western blot (exemplary blot in (**b**)). β-actin (48 kDa) expression was used for normalization. Band volumes were calculated in TotalLab (**a**) in relation to the untreated control. Data were normally distributed and one-sample *t*-test was conducted. * *p* ≤ 0.05 (compared to co, no PIC); co = control, *n* = 5.

Further tasks of RPE cells involve recycling of visual pigments with RPE65 as an important enzyme [47], and the regulation of the complement system on the basolateral site by expression of CD59 [48]. Protein expression of RPE65 (Figure 8) and CD59 (Figure 9) in primary RPE was tested by Western blotting. RPE were stimulated with 50 µg/mL FucBB04 and/or 10 µg/mL PIC for three days. Band volumes were evaluated and set into relation to control without PIC. Example blots are shown in Figure 8b and Figure 9b.

PIC significantly reduced RPE65 expression (Figure 8a) to 0.6 ± 0.5 [arb. unit] (*p* = 0.0002). FucBB04 alone significantly reduced RPE65 to 0.5 ± 0.2 [arb. unit] (*p* = 0.0033) and to 0.3 ± 0.1 [arb. unit] (*p* = 0.0003) when co-applied with PIC. Regarding CD59 (Figure 9b), PIC significantly reduced its expression to 0.8 ± 0.3 [arb. unit] (*p* = 0.0464). FucBB04 displayed no significant influence on CD59 expression. Of note, the lowering effect of PIC on CD59 expression was lost under FucBB04 stimulation.

### 2.8. Phagocytosis Assay

**Figure 10:**
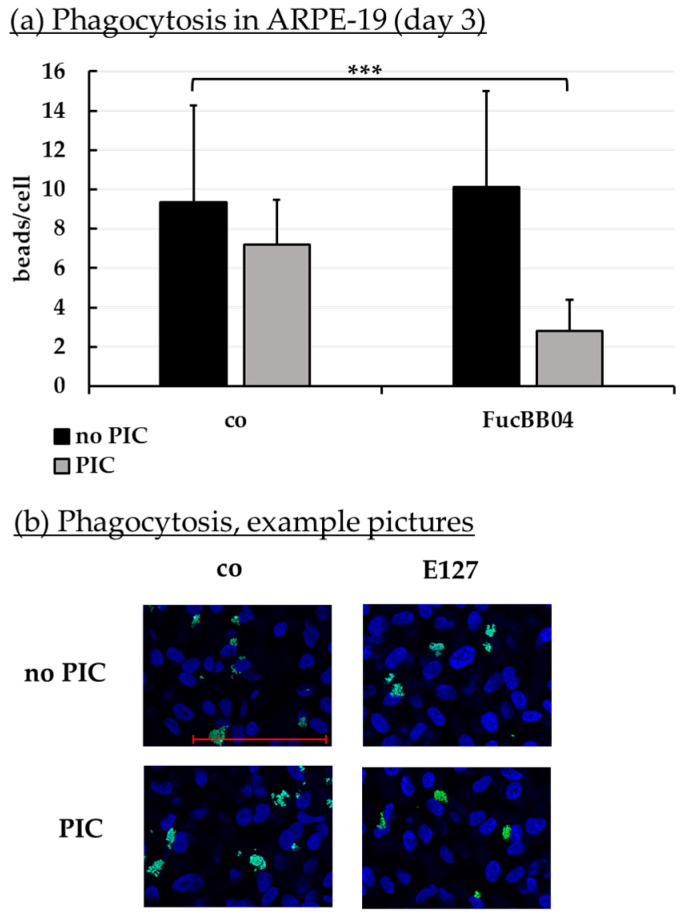
Phagocytosis of ARPE-19. ARPE-19 cells were stimulated with 50 µg/mL FucBB04 and/or 10 µg/mL polyinosinic/polycytidylic acid (PIC) for three days. Fluorescence-labeled beads opsonized with photoreceptor outer segments were applied to the cells (green dots, **b**). Fluoromount-G™ and 4′,6-diamidino-2-phenylindol (DAPI) was used to label cell nuclei (blue circles, **b**). Immunofluorescence photos were taken by Imager M2 (64× magnification, red scale bar = 100 µm). Nuclei number and beads were counted. Beads were calculated relatively to cell nuclei (**a**). Shapiro–Wilk test showed Gaussian distribution. Data were normally distributed and one-sample *t*-test was conducted. *** *p* ≤ 0.001 (compared to control, no PIC), co = control, *n* = 15.

**Figure 11:**
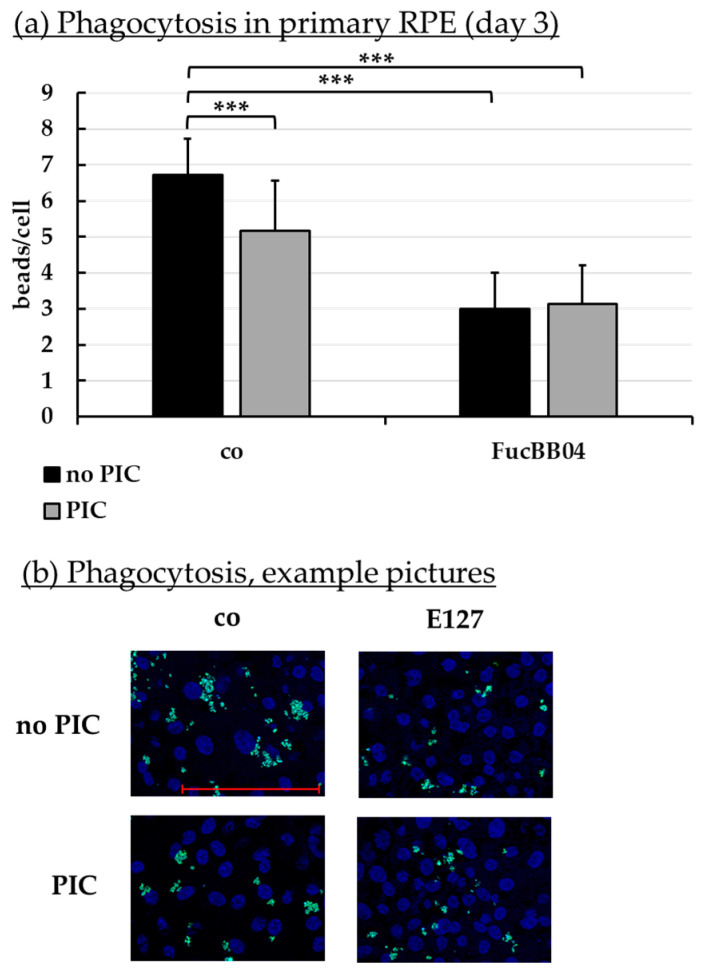
Phagocytosis of RPE. Primary porcine RPE cells were stimulated with 50 µg/mL FucBB04 and/or 10 µg/mL polyinosinic/polycytidylic acid (PIC) for three days. Fluorescence-labeled beads opsonized with photoreceptor outer segments were applied to the cells (green dots, **b**). Fluoromount-G™ and 4′,6-diamidino-2-phenylindol (DAPI) was used to label cell nuclei (blue circles, **b**). Immunofluorescence photos were taken by Imager M2 (64× magnification, red scale bar = 100 µm). Nuclei number and beads were counted. Beads were calculated relatively to cell nuclei (**a**). Shapiro–Wilk test showed Gaussian distribution. Data were normally distributed and one-sample *t*-test was conducted. *** *p* ≤ 0.001 (compared to control, no PIC), co = control, *n* = 15.

Phagocytosis is essential for photoreceptor outer segment renewal and visual pigment recycling [4]. Human RPE cell line ARPE-19 (Figure 10) and primary porcine RPE (Figure 11) were treated with 50 µg/mL FucBB04 and/or 10 µg/mL PIC for three days. Phagocytosis was assessed with fluorescent latex beads. Number of ingested beads were put in relation to cell nuclei. Exemplary photos are shown in Figure 10b and Figure 11b. In ARPE-19, the co-stimulation with FucBB04 and PIC significantly reduced phagocytosis (Figure 10a) from 9 ± 5 beads/cell to 3 ± 2 beads/cell (*p* = 0.0002). Neither pro-inflammatory activation by PIC alone nor FucBB04 alone displayed a significant effect on phagocytosis. Concerning primary RPE, FucBB04 significantly reduced phagocytosis (Figure 11a) from 7 ± 3 beads/cell to 3 ± 1 beads/cell (*p* < 0.0001). Also, PIC and co-treatment with FucBB04 and PIC reduced RPE phagocytosis to 5 ± 1 beads/cell (*p* = 0.0268) and to 3 ± 1 beads/cell (*p* < 0.0001), respectively.

## 3. Discussion

In our study, we purified a very high-molecular weight fucoidan (3700 kDa) from *Laminaria hyperborea* and tested its bioactivity in regard to a potential application for the treatment of AMD. While this fucoidan showed some interesting bioactivities, its influence on RPE cell function renders it not suitable for further development in AMD treatment.

The chemical characterization of fucoidan sample FucBB04 revealed the highest molecular weight average for fucoidan yet reported (3700 kDa), likely achieved through the mild extraction and purification methods as well as ultrafiltration applying 300 kDa MWCO filters, deliberately removing, beside other co-extracted seaweed polysaccharides such as laminarin or alginate, lower molecular weight fucoidan fractions [35]. The predominant sugar moiety is fucose with 91.59%. The rms conformation plot revealed a molecular shape indicative of polymeric branching or hyper-branching, respectively [39,40]. Considering previously reported structural elucidations [20,33], a fucoidan backbone structure remains likely, with polymeric side-chains containing sugar moieties such as rhamnose, xylose, and uronic acids, previously not detected in fucoidan from LH. However, the branching pattern and sugar moiety distribution is currently speculative and requires further experiments such as branching analysis via SEC-MALS, target-specific depolymerization using fucoidan degrading enzymes, selectively cleaving glycosidic linkages and sulfate groups [49,50], and/or mild hydrolysis followed by fractionation and subsequent analysis of the molecular segments.

The bioactivities of fucoidans are strongly related to their molecular properties, of which molecular weight is of high importance. For example, molecular weight can make the difference in pro- and antiangiogenic properties of fucoidan [17], can influence the antibacterial effect [51], the skin-whitening effect [52], or the anticoagulant effect of fucoidans [53].

Fucoidans have been described to exert antioxidative effects; however, the relation between structure and antioxidant activity is complex and some of their antioxidant behavior has been attributed to contaminant phenol contents [54,55]. FucBB04 did not exert any antioxidative protective effect in our tested cell culture models, which on the other hand was shown for a 1549 kDa LH fucoidan [20,42]. This LH fucoidan was slightly protective in OMM-1 against H_2_O_2_ [20], showed promising antiferroptotic activities, and increased GPX4 expression in both OMM-1 and ARPE-19 [42]. In our experience, the protective effect against oxidative stress of fucoidans is not very prominent in retinal pigment epithelial cell models, but is more easily determined in OMM-1 cells, as RPE cells are rather resistant to oxidative stress with a strong intrinsic protective response [56]. As FucBB04 did not exert any effect in OMM-1 cells, its oxidative stress protection can be considered negligible in the ocular context.

Fucoidans can exhibit antiangiogenic and VEGF-inhibiting effects which make them highly interesting for conditions such as AMD or diabetic retinopathy [12]. FucBB04 inhibited VEGF after seven days in primary porcine RPE cells at a similar concentration and time frame that has been shown to effectively reduce VEGF in other fucoidans [43]. Conversely, however, FucBB04 exhibited no VEGF-inhibiting effect in ARPE-19 cells. The lack of effect on ARPE-19 is surprising, as in our experience, VEGF inhibition is easier achieved in ARPE-19 than in primary porcine RPE cells, as RPE cells secrete much higher concentrations of VEGF than ARPE-19 per time unit [26]. Also, the VEGF inhibition in primary RPE cells did not reach the potency of the 1549 kDa LH fucoidan [20], rendering it inferior in VEGF inhibition compared to other tested fucoidans. Of note, the VEGF-reducing effects are only seen in lower concentrations of FucBB04. Similar results have been found in a different fucoidan regarding inflammation, with lower concentrations of fucoidan showing the strongest effect [57]. As fucoidans have been shown to bind simultaneously to VEGF and its receptor [58], inhibiting angiogenic signaling and potentially reducing VEGF expression this way [59,60], it is feasible that a higher concentration of fucoidan may sterically hinder this interaction or may reduce the probability of a fucoidan molecule binding VEGF and its receptor at the same time due to the abundance of the fucoidan-binding partner.

Inflammation is an important factor in the development of AMD [9], and RPE cells secrete pro-inflammatory cytokines after stimulation with a variety of pro-inflammatory insults [7]. Interestingly, the effect of fucoidans on cytokine secretion is dependent on the stimulus, indicating an interaction with the TLR-3 pathway [14,43]. FucBB04 displayed a similar influence, with reducing IL-6 and IL-8 after PIC treatment (3 and 7 days, respectively), with some effect on IL-8 secretion also seen after LPS stimulation (after 1 and 28 days). Taken together, the effect of FucBB04 on IL-6 and IL-8 secretion is comparable with that of other fucoidans, suggesting mainly an interaction with the TLR-3 pathway. RPE cells highly express TLR-3, and its activation does not only induce pro-inflammatory cytokine secretion, but also a reduction of RPE65 expression and phagocytosis, thereby reducing RPE function before inducing cell death [8,61]; however, the pathways leading to RPE65 and phagocytosis reduction are not elucidated so far. It would be feasible to suppose that the reduction of RPE65 and phagocytosis may be a consequence of a sub-lethal injury of the cell, rending them alive but no longer functional, as described by Spaide et al. [62].

The nature of this interaction between the TLR-3 pathway and fucoidans is not known so far, but could be mediated via a steric interaction with the TLR-3 pathway. As TLR-3 activation has been implicated to be involved in AMD-development [8,63,64,65], this stresses again the potential of fucoidans in preventive or early treatment of AMD. However, it should also be noted that FucBB04 significantly induced IL-8 after 1 day of stimulation in the absence of a pro-inflammatory stimulus, albeit to a lesser degree compared to pro-inflammatory stimuli. Indeed, fucoidans have been shown to induce cytokine release, thereby again stressing the importance of meticulous characterization of any fucoidan considered for potential therapeutic use [43].

The function of the RPE is vital for the survival of the photoreceptors and the maintenance of visual function. One important function is the phagocytosis of shed photoreceptor outer segments, which in vivo are shed every morning after the onset of light [4]. Indeed, impairment of RPE phagocytosis leads to blindness in animal models and patients [66,67]. In our study, FucBB04 significantly and relevantly reduced the phagocytic capabilities of RPE cells. Fucoidans have been shown before to be able to reduce phagocytosis of activated macrophages [68,69]. Indeed, we have recently shown that FucBB04 reduces the phagocytotic capabilities of retinal microglia [70]. However, the mechanisms of phagocytosis of macrophages differ from those of RPE cells [71]. In macrophages, fucoidan has been described to alter phagocytosis by modulating TNF production of peripheral blood mononuclear cells or by interfering with the scavenger receptor [68,69]. We have previously tested the effect of several fucoidans on phagocytotic activity. Commercially available fucoidan from *Fucus vesiculosus* (FV) did not impede phagocytic function in primary RPE cells [14,59]. On the other hand, fucoidan from a crude extract of *Fucus distichus* subspecies *evanescens* reduced phagocytosis in primary RPE cells [72]. Of interest, we have investigated the effect of a high-molecular weight fucoidan (1549 kDa) from LH which did not show a significant influence on phagocytosis in the same experimental setting [34], strongly indicating that the effect is molecular weight dependent. As the phagocytosis of photoreceptor outer segment fragments of the RPE is dependent on the interaction of αvβ5 integrin with binding partners like lactadherin (MFGE8), the mechanism of inhibition could be a steric inhibition of this interaction by the large, negatively charged fucoidan [73].

Another vital function of the RPE is to contribute to the recycling of the visual pigment to uphold vision. An important enzyme in this process is the retinyl ester-binding protein RPE65. Lack of functional RPE65 can lead to blinding disease, such as retinitis pigmentosa or Leber’s congenital amaurosis and has been successfully targeted in gene-therapy [74,75]. FucBB04 significantly and relevantly reduced the expression of RPE65 in primary RPE cells. We have previously tested the effect of LH fucoidan with a molecular weight of 1549 kDa on RPE65 expression, and no effect could be found [34]. This strongly suggests that the effect on RPE65 expression is molecular weight dependent. However, a study concerning different fucoidans from *Saccharina latissima* (SL) shows that a fucoidan from the Faeroe Islands with a molecular weight of 997 kDa does not influence RPE65 expression, while a Norwegian SL extract with a molecular weight of 312 kDa reduced RPE65 [43]. Of interest, commercially available fucoidan from FV did not influence RPE65 expression but protected RPE cells from RPE65 loss after pro-inflammatory stimulation [14] (this protection was not seen with SL fucoidans [43]). We do not know why and how some fucoidans reduce RPE65 expression, but this effect should be tested for fucoidans scheduled for further development as a potential AMD therapeutic in order to avoid any interaction with RPE65 expression.

Also of importance is the outer blood–retinal barrier, which contributes to the immune privilege of the retina [6]. FucBB04 transiently reduced the barrier properties of primary RPE cells one day after stimulation. In addition, it exacerbated the negative effect of TNF on the cell barrier. This effect was not found with FV fucoidan (which ameliorated PIC-induced barrier reduction after 28 days) [14], but is similar to what is seen for two SL fucoidans, which also reduced barrier function after 1, but not after 3 days [43]. A transient reduction in the barrier function may not be of a high biological relevance. However, the exacerbation of pro-inflammatory effects would argue against an application in AMD, as inflammation is considered to be an important pathomechanism.

Our study has several limitations. It is an *in vitro* study using cell culture models. While this gives us valuable insight into cellular effects and mechanisms, it cannot reflect on the complex interactions that are seen in vivo. In particular, cancer cell lines like OMM-1 are highly abnormal compared to normal human tissue. In addition, ARPE-19 cells, while not derived from a tumor, only partially behave as RPE cells. Finally, while primary porcine RPE cells are an excellent model of adult human RPE cells, they lack the interaction with the adjacent cells and tissues of the eye and are of animal origin [24].

Taken together, our results show some protective bioactivities of FucBB04 such as a reduction of TLR-3-mediated cytokine release and VEGF inhibition, but also negative influences regarding RPE function such as a reduction of phagocytosis and RPE65 expression. As these negative effects are not found with a 1549 kDa LH fucoidan, we would advise against the use of very high-molecular weight fucoidan for further development of AMD therapeutics.

## 4. Materials and Methods

### 4.1. Algal Material, Fucoidan Extraction and Purification Method

*Laminaria hyperborea* (Laminariaceae, Phaeophyceae) were harvested on 6 April 2020 along the coast of Haugesund, Norway (Rogaland field 55E; N 59°11′ E 005°06′). A mild warm water extraction under stirring was performed for one hour at 60 °C, using freshly harvested, shredded LH leaves. The liquid phase was separated and CaCl_2_ added. After one hour of settling, a centrifugation followed by microfiltration (at 100, 50, and 20 µm pore size, consecutively) was performed to remove remaining algae particles and precipitated alginate. Salt and smaller organic molecules were then removed through ultrafiltration using 300 kDa MWCO ceramic filters and 0.1 mol/L NaCl solution followed by distilled water. Fucoidan in the filtrate was precipitated using ethanol. Excess liquid was removed and the precipitated fucoidan vacuum dried.

### 4.2. Molecular Weight Determination

The molecular weight of FucBB04 was determined through size-exclusion chromatography (SEC) in the form of HPLC equipped with online multiangle static light scattering (MALS). The measurements were performed at ambient temperature using a Shodex LB-806 and LB-805, as well as a 2500 PWXL column as a separator. The measurement was executed with a Dawn HELEOS-8+ multiangle laser light scattering photometer (Wyatt, Santa Barbara, CA, USA) (λ0 = 660 nm) and a subsequent Optilab T-rEX differential refractometer. The mobile phase was 0.15 mol/L CH_3_COONH_4_ (ammonium acetate at pH = 7) with 0.01 mol/L EDTA added. The flow rate was 0.5 mL/min. The injection volume was 5 µL, with a concentration of 5 g/L. The data were obtained and processed using Astra (v. 7) software (Wyatt, Santa Barbara, CA, USA).

### 4.3. Sulfate Content

The sulfur content was determined through an Agilent 7500 Series quadrupole ICP-MS. The fucoidan was dried at 70 °C for 90 min and 5.0 mg dissolved in 15.0 mL of 1 M HNO_3_. The sulfation degree was calculated by using a mass balance equation, assuming that every sulfate group is associated with a sodium counterion. The sulfate position was verified via Raman micro-spectroscopy. Measurements were performed at room temperature using a Bruker Senterra II Spectrometer equipped with a 785 nm laser and scanned from 100 to 4000 cm^−1^ (integration time 30 s, 3 accumulations). A sample size of <0.5 mg gave sufficient resolution due to the microscope array. No further sample preparation was necessary.

### 4.4. Total Phenolic Content

The total phenolic content (TPC) was determined using the Folin–Ciocalteu TPC assay applying a method optimized for seaweeds, as described in Wekre et al. (2022) [76,77]. Briefly, the method used 0.2 mL sample, blank, or standard, 1.59 mL undiluted Folin–Ciocalteu reagent, 4.0 mL 20% (*w*/*v*) Na_2_CO_3_ and yielded a total volume of 20 mL with water. The mixture was incubated for two hours in the dark, and absorbance was measured at 760 nm using a Hitachi Model U-5100 UV/Vis Spectrophotometer (Hitachi, Tokyo, Japan). Gallic acid and phloroglucinol calibration curves were used to validate the linearity, sensitivity, precision, and accuracy of the TPC method.

### 4.5. Monosaccharide Analysis

To analyze the monosaccharides composition, 1 mg of fucoidan was treated with 2 M trifluoroacetic acid (TFA) at 120 °C for three hours. After centrifugation at 10,000 rpm for ten minutes, 100 μL of supernatant was vacuum dried and resuspended in H_2_O. Samples were then analyzed through high-performance anion-exchange chromatography with pulsed amperometric detection on an IC6000 system operated by Chromeleon software version 7.3 (Dionex, Sunnyvale, CA, USA). Analysis was conducted using Dionex CarboPac PA1 (4 × 250 mm) column at 20 °C and flow rate of 1 mL/min. Eluent A (water), B (300 mM NaOH), C (200 mM NaOH in 170 mM NaOAc), and D (1 M NaOAc) were prepared for the separation of uronic acids. All samples were prepared in triplicates. Monosaccharides were identified and quantified by comparing their retention times and peak areas to standards of known concentrations. The standards used were L-guluronic acid and D-mannuronic acid (purchased from Carbosynth, Berkshire, UK), while fucose, rhamnose, galactose, glucose, mannose, and xylose (purchased from Sigma-Aldrich, St. Louis, MO, USA) were dissolved in H_2_O and run in different concentrations ranging from 0.005 g/L to 0.1 g/L. Water was used to elute natural sugars while a gradient was solely used to elute uronic acids.

### 4.6. Cell Culture

OMM-1 (RRID: CVCL_6939) cell line from human uveal melanoma [23] was kindly provided by Dr. Sarah Coupland. Hundred µL of cells at concentration of 200,000 cell per mL were seeded in 96-well plate (Sarstedt, Nümbrecht, Germany; #83.3924.005) and treated after 24 h of incubation at around 90% confluence, as media RPMI 1640 (Pan-Biotech, Aidenbach, Germany; #P04-18500) with 10% fetal bovine serum (Thermo Fisher Scientific, Waltham, MA, USA; #A5256701), and 1% penicillin/streptomycin (Sigma-Aldrich, St. Louis, MO, USA; #P0781) were used. Cells were split 1:4 after 3–4 days.

Immortal ARPE-19 cell line, originally from a 19-year-old human donor [21], was purchased from American Type Culture Collection (ATCC, Manassas, VA, USA; RRID: CVCL_0145; #CRL-2302). These cells were seeded in 12-well (1 mL per well, Sarstedt; #83.3921) or 96-well plates (100 µL per well) with 100,000 cells per mL and treated after one week in 12-well plates or after 24 h in 96-well plates (90% confluence), as media HyClone DMEM (Cytiva, Freiburg in Breisgau, Germany; #31053028) with 10% fetal bovine serum, 1% penicillin/streptomycin, 2.5% HEPES (Pan-Biotech; #P05-01100), and 1% non-essential amino acids (Pan-Biotech; #P08-32100) were used.

Primary RPE cells from pig eyes were isolated as described before [78]. Eyes were delivered from local slaughters as byproduct in food production. The use of these eyes was agreed to by animal welfare officer of University of Kiel. It is considered an active contribution to the reduction in animal experiments (German animal welfare act TierSchG) according to the 3R principle. The eyes were cleaned and cut open to remove retina and vitreous body. RPE cells were removed by incubation steps with trypsin (Pan-Biotech; #P10-021100) and trypsin/EDTA (Pan-Biotech; #P10-020100). After washing, cells were seeded into 12-well (1 mL per well) or 24-well (500 µL per well; #83.3922.005) plates with 100,000 cells per mL, as media HyClone DMEM with 10% fetal bovine serum, 1% penicillin/streptomycin, 2.5% HEPES, and 1% non-essential amino acids were used. Cells were always treated at 100% confluence (two weeks after seeding). To measure TEER, cells were seeded on 12-well plates containing a transwell insert (Sarstedt; #83.3931.041).

For phagocytosis assay, ARPE-19 and RPE cells were seeded on cover slips (Th. Geyer GmbH & Co. KG, Renningen, Germany; #9161050) which were coated with collagen I, also called collagen A (Pan-Biotech; #P06-20030), according to the manufacturer’s instructions.

Cell cultures were incubated at 37 °C and 5% CO_2_ in a humidified incubator. In general, cells were fed two times per week, also during long-term stimulation experiments for RPE (media were supplied with stimuli).

### 4.7. Stimulation

Cells were treated with 1–100 µg/mL FucBB04 depending on the experiment. For oxidative stress assays, ARPE-19 were treated first with FucBB04 for 30 min, followed by 250 µM H_2_O_2_ (Sigma-Aldrich; #H1009) and 20 µM erastin (Cayman Chemical, Ann Arbor, MI, USA; #CAY17754-5) for 24 h; OMM-1 were treated with 500 µM H_2_O_2_ or 25 µM erastin for 4 h after 30 min of fucoidan incubation, both applied in MTT assay (refer to Section 4.8). For VEGF assays, ARPE-19 were treated with FucBB04 for three days, followed by supernatant collection and MTT assay. Primary RPE were treated with fucoidan for 28 days, harvesting supernatants on day 3, 7, and 28 from the same well. The experiment was terminated by an MTT assay on day 28. For inflammation assays, cells were treated with FucBB04 for 30 min followed by a pro-inflammatory stimulus for 28 days with supernatant collection on day 1, 3, 7, and 28 days from the same well, finalizing with MTT assay on day 28 (1 µg/mL LPS (Merck, Darmstadt, Germany; #L4524), 10 µg/mL PIC (Tocris Bioscience, Bristol, UK; #4287/10), 50 ng/mL TNF (R&D System, Minneapolis, MN, USA; 210-TA-005/CF), or 10 ng/mL Pam2CSK4 (Pam, Tocris Biosciences; #4637)). For both VEGF and inflammation assays, RPE cells were re-stimulated after three to four days, as well as after supernatant harvest (refer to Section 4.10).

### 4.8. Cell Viability

MTT assay was used to assess cell viability and oxidative stress protection [79]. Wells were washed with phosphate-buffered saline (PBS, Pan-Biotech; #P04-53500) and then 0.5 mg/mL MTT solution (Sigma-Aldrich; #M2128) in HyClone DMEM without phenol red (Cytiva; #SH30284.01) was applied for two hours. MTT was discarded and cells lysed with dimethyl sulfoxide (Carl Roth, Karlsruhe, Germany; #11657618). Readout was performed at 550 nm by using the Elx800 (BioTek, Bad Friedrichshall, Germany).

### 4.9. Transepithelial Electrical Resistance (TEER)

For TEER measurements, primary porcine RPE were cultivated in 12-well plates with transwell inserts. TEER was measured with Epithelial Volt/Ohm (EVOM™) Meter 3 with STX4 electrodes (WPI, Sarasota, FL, USA). Between measurements, electrodes were washed in 70% ethanol (Th. Geyer GmbH & Co. KG; #11655285) and sterile distilled water (Fresenius Kabi AG, Bad Homburg, Germany; #1080224). TEER values were documented 7, 10, and 14 days after preparation. A well without cells and with media was used as blank. Only wells with a TEER of at least 150 Ohm·cm^2^ were included in the study [80]. TEER was measured before stimulation (day 0) and after 1 and 3 days after stimulation.

### 4.10. Enzyme-Linked Immunosorbent Assay

**Figure A1:**
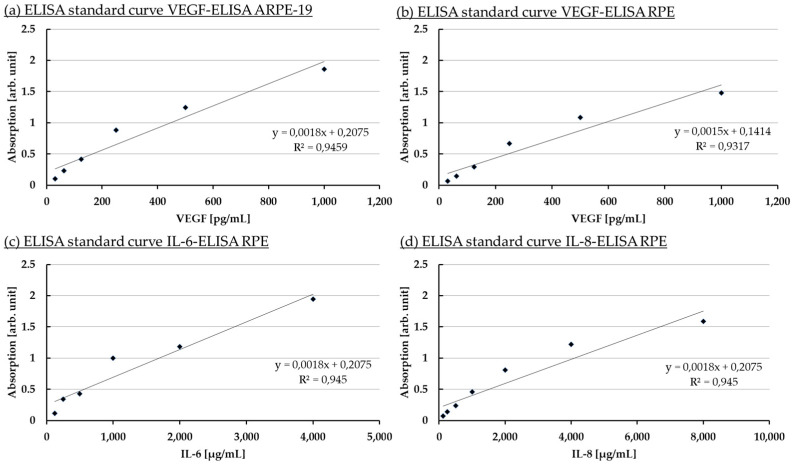
Standard calibration curves. Standard curves for Section 2.3 (VEGF-ELISA ARPE-19 and RPE) and Section 2.4 (IL-6-ELISA and IL-8-ELISA) are shown.

ELISA was used to assess VEGF, IL-6, and IL-8 in supernatants. These were collected after indicated stimulation times. Supernatants were collected for 24 h (ARPE-19: VEGF, RPE: IL-6, IL-8) or 4 h (RPE: VEGF). ELISA DuoSet for human VEGF was used to determine VEGF-A of ARPE-19 and RPE samples. ELISA DuoSets for porcine IL-6 and IL-8 were used for RPE samples, all bought from R&D Systems (Minneapolis, MN, USA; # DY293B; #DY686; #DY535). All kits were used according to the manufacturer’s instructions. Standard calibration curves for all ELISA are shown in Appendix A.2 (Figure A1).

### 4.11. Real-Time Polymerase Chain Reaction

RNA was isolated with NucleoSpin RNA Mini Kit (Macherey-Nagel, Düren, Germany; #11699517) according to the manual. Genomic DNA was digested with DNase, provided by the manufacturer. RNA was dissolved in 20 µL RNase-free water from the kit. NanoDrop™ One (Thermo Fisher Scientific) was used to analyze purity and concentration of the RNA samples. To transcribe RNA into cDNA, the High-Capacity cDNA Reverse Transcription Kit (Thermo Fisher Scientific; #4368814) was used according to the manual. Real-time PCR was conducted with TaqMan™ Fast Advanced Master Mix (Thermo Fisher Scientific; #4444557) and TaqMan™ gene expression assays (Thermo Fisher Scientific; #4331182) [dye label 5(6)-carboxyfluorescein-minor groove binder (FAM-MGB)]. The procedure was performed as described in the instructions of the TaqMan™ Fast Advanced Master Mix. Gene expression assays for *IL6* (interleukin 6, Ss03384604_u1), *CXCL8* (interleukin 8, Ss03392437_m1), and *GAPDH* (glyceraldehyde-3-phosphate dehydrogenase, Ss03375629_u1, endogenous control) were used. For evaluation, RQ module of Thermo Fisher Connect was used which is based on ΔΔCT method [81]. The ΔΔCT method calculated the relative normalized gene expression with ΔCT (=CT [gene of interest] − CT [housekeeping gene]), ΔΔCT (=ΔCT [sample of interest] − ΔCT [reference sample]), and relative fold gene expression level RQ (=2^−ΔΔCT^). Reference sample was PIC and set to RQ = 1.0.

### 4.12. Western Blotting

To assess RPE65 and CD59 protein expression, Western blots were conducted as previously described [43]. In brief, after treatment, cells were washed in PBS and lysed with Nonidet^®^ P-40 containing lysis buffer (NP-40, nonylphenyl-polyethylene glycol, Sigma-Aldrich; #11332473001) for 45 min on ice. DC Protein Assay Kit II (Bio-Rad Laboratories, Munich, Germany; #5000112 and Genesys 10 Bio (Thermo Fisher) were used to determine protein concentrations as described by the manufacturer. SDS-PAGE was performed with 12% acrylamide gels and 15 µg sample. Wet tank Western blots were performed and membranes blocked with 4% skimming milk (Carl Roth GmbH + Co. KG, Karlsruhe, Germany; #T145.2). Membranes were incubated overnight at 4 °C with primary antibodies in 2% skimming milk (mouse anti-RPE65 Abcam, 65 kDa, 1:6000, Berlin, Germany; #ab235950; rabbit anti-CD59, 18 kDa, 1:3000, Proteintech Group, Inc., Rosemont, IL, USA; #10742-1-AP; rabbit anti-β-actin, 37 kDa, 1:1000, Cell Signaling Technologies, Denver, CO, USA; #4967). Conjugates with horseradish peroxidase (HRP) were applied for one hour (anti-mouse-HRP or anti-rabbit-HRP, Cell Signaling Technologies; #7076, #7074). For chemiluminescent signal, Amersham ECL Western Blotting Detection Reagent (GE Healthcare, Chicago, IL, USA; #12316992) was applied. Readout was performed with MF-ChemiBIS 1.6 (Biostep, Jahnsdorf, Germany). TotalLab TL100 (Biostep, version 1D v2008) software was used to generate quantitative values and β-actin was used for normalization

### 4.13. Phagocytosis Assay

Confluent RPE cells on collagen I-coated cover slips were treated and used for the phagocytosis assay (conducted as previously established [82]). Latex fluorescence beads (Sigma-Aldrich; #L4530) were incubated with photoreceptor outer segment fragments, prepared from porcine eyes‘ retinae as described previously [72]. The treated beads were applied to the respective cells for four hours. After washing cells with media and PBS, they were fixated in paraformaldehyde (Carl Roth GmbH + Co. KG; #0335.1) and mounted with Fluoromount-G™ with DAPI (Thermo Fisher Scientific; #00-4959-52). Images were taken with fluorescence microscope Axiovert Imager M.2 by using ZenBlue 2 Software (version) v2.0.0.0) (both Zeiss, Jena, Germany). Cell nuclei and beads were counted by self-programmed macro in Fiji (ImageJ2, https://imagej.net/software/fiji/downloads (accessed on 29 March 2021)). Counted beads were calculated in relation to the cell nuclei number.

### 4.14. Statistical Analysis

Microsoft Excel and PowerPoint (Microsoft Office 2010, Microsoft, Redmond, WA, USA) were used to generate diagrams depicting means and standard deviations. GraphPad Prism 9 (GraphPad Software, Inc., San Diego, CA, USA, version 9.1.1) was used for statistical evaluation. Normality was assessed with Shapiro–Wilk test. One-sample *t*-test was used for calculating significances between relative data and ANOVA (analysis of variance) followed by Dunnett’s multiple comparison test was used for absolute data. PCR data were evaluated by Thermo Fisher Connect, which uses the student’s *t*-test. Bio groups with *p*-values ≤ 0.5 were marked as significant.

## 5. Conclusions

The aim of this study was to assess the bioactivity of a very high-molecular weight (3700 kDa) fucoidan from *Laminaria hyperborea* from the coasts of Norway, concerning age-related macular degeneration-relevant pathomechanisms and their influence on retinal pigment epithelium cell functions. In former studies, another fucoidan from this species with a size of 1549 kDa showed antioxidative, antiangiogenic, and anti-inflammatory effects with no adverse effects on retinal pigment epithelium cell functions. With the bigger fucoidan from this study, we also determined vascular endothelial growth factor-inhibiting effects and a reduction in interleukin 6 and interleukin 8 secretion. Antioxidative effects were not found. Barrier function was transiently affected. Retinal pigment epithelium 65 kDa protein expression as well as phagocytic ability were reduced. Taken together, we determined antiangiogenic and anti-inflammatory effects in primary retinal pigment epithelium, but the effects on cellular functions advise against further development of this very high-molecular weight fucoidan from *Laminaria hyperborea* as a potential therapeutic in age-related macular degeneration.

## Figures and Tables

**Table 1:** Monosaccharide composition of very high-molecular weight fucoidan from *Laminaria hyperborea*. Analysis performed in three replicates (molecular percentage and standard deviation are indicated). Fuc = fucose; Rha = rhamnose; Gal = galactose; Xyl = xylose; Man = mannose; GalA = galacturonic acid; ManA = mannuronic acid; GulA = guluronic acid; SD = standard deviation.

Sugar	Fuc	Rha	Gal	Xyl	Man	GalA	ManA	GulA	Other
Mol%	91.59	1.32	0.50	0.71	0.44	1.60	1.95	1.86	0.53
SD	1.40	0.26	0.42	0.05	0.15	0.12	0.23	0.08	–

**Table 2:** Data overview of the sulfated fucan samples used in this study. Degree of sulfation (DS), weight average molar mass (Mw), number average molar mass (Mn), polydispersity index (PD), degree of polymerization (DP_n_), Z-average radius of gyration (Rz), refractive index increment (dn/dc), slope of the RMS conformation plot (b = rms versus M), and the total phenolic content in the sample (TPC).

Fucoidan	DS	M_w_ [kDa]	M_n_ [kDa]	PD	DP_n_	R_z_ [nm]	dn/dc	B	TPC [%]
FucBB04	0.95	3700	2500	1.48	10,300	249	0.115	0.44	0.0

**Table 3:** Inflammatory gene expression (three days). Real-time polymerase chain reaction (qPCR) was used to determine gene expression of *IL6* (interleukin 6) and *CXCL8/IL8* (interleukin 8) in primary porcine RPE. *GAPDH* was used as endogenous control. Cells were treated with 50 µg/mL FucBB04 and/or 10 µg/mL polyinosinic/polycytidylic acid (PIC) for three days. Thermo Fisher Connect was used to determine relative gene expression and significances (student’s *t*-test). co = untreated control, Rq = relative fold gene expression level (= 2^−ΔΔCT^), Rq Min = minimal Rq value, Rq Max = maximal Rq values. No significant findings, *n* = 3.

Bio Group Name	Target Name	Rq	Rq Min	Rq Max	*p*-Value
control	*IL6*	1.649	0.305	8.916	0.661
PIC	*IL6*	1.000	0.725	1.379	1.000
FucBB04	*IL6*	0.574	0.156	2.110	0.540
FucBB04+PIC	*IL6*	0.603	0.157	2.321	0.586
control	*CXCL8*	0.434	0.318	0.593	0.162
PIC	*CXCL8*	1.000	0.498	2.007	1.000
FucBB04	*CXCL8*	0.872	0.411	1.852	0.829
FucBB04+PIC	*CXCL8*	1.165	0.539	2.518	0.811

**Table 4:** Inflammatory gene expression (seven days). Real-time polymerase chain reaction (qPCR) was used to determine gene expression of *IL6* (interleukin 6) and *CXCL8/IL8* (interleukin 8) in primary porcine RPE. *GAPDH* was used as endogenous control. Cells were treated with 50 µg/mL FucBB04 and/or 10 µg/mL polyinosinic/polycytidylic acid (PIC) for three days. Thermo Fisher Connect was used to determine relative gene expression and significances (student’s *t*-test). co = untreated control, Rq = relative fold gene expression level (= 2^−ΔΔCT^), Rq Min = minimal Rq value, Rq Max = maximal Rq values. No significant findings, *n* = 3.

Bio Group Name	Target Name	Rq	Rq Min	Rq Max	*p*-Value
control	*IL6*	0.570	0.127	2.550	0.586
PIC	*IL6*	1.000	0.694	1.442	1.000
FucBB04	*IL6*	1.671	1.393	2.006	0.120
FucBB04+PIC	*IL6*	0.854	0.446	1.633	0.736
control	*CXCL8*	0.270	0.242	0.301	0.134
PIC	*CXCL8*	1.000	0.393	2.544	1.000
FucBB04	*CXCL8*	0.493	0.251	0.969	0.353
FucBB04+PIC	*CXCL8*	1.847	0.326	10.475	0.627

## Data Availability

Data are available on request.

## Appendix A

### Appendix A.1. Cell Viability Data for Section 2.5

In this section, supporting cell viability data determined by MTT assay for inflammation assays of Section 2.5 are listed (Table A1). Viability was determined 1, 3, 7, and 28 days after stimulation with FucBB04 and pro-inflammatory agents. There were no significant, relevant findings observed.

**Table A1:** Cell viability data with mean and standard deviation (SD). LPS = lipopolysaccharide, PIC = polyinosinic/polycytidylic acid, TNF = tumor necrosis factor alpha, Pam = Pam2CSK4 Pam.

Day 1	co	LPS	PIC	TNF	Pam	
no FucBB04	100	105	109	104	103	Mean
	0	11	14	32	9	SD
FucBB04	95	91	94	96	97	Mean
	3	7	7	5	1	SD
**Day 3**						
no FucBB04	100	94	96	105	99	Mean
	0	15	7	9	5	SD
FucBB04	98	87	77	95	96	Mean
	4	16	15	1	1	SD
**Day 7**						
no FucBB04	100	97	95	93	100	Mean
	0	13	14	18	12	SD
FucBB04	100	81	88	98	91	Mean
	6	9	5	15	9	SD
**Day 28**						
no FucBB04	100	90	98	99	81	Mean
	0	19	26	44	31	SD
FucBB04	75	64	78	90	63	Mean
	16	16	16	13	15	SD

### Appendix A.2. Standard Calibration Curves for ELISA

In this section, standard calibration curves for Section 2.3 (VEGF-ELISA ARPE-19 and RPE) and Section 2.4 (IL-6-ELISA and IL-8-ELISA) are shown (Figure A1).
